# Influence of Prosulfocarb and Polymer Supplementation on Soil Bacterial Diversity in *Triticum aestivum* L. Cultivation

**DOI:** 10.3390/ijms26125452

**Published:** 2025-06-06

**Authors:** Małgorzata Baćmaga, Jadwiga Wyszkowska, Jan Kucharski

**Affiliations:** Department of Soil Science and Microbiology, Faculty of Agriculture and Forestry, University of Warmia and Mazury in Olsztyn, Łódzki 3 Sq., 10-719 Olsztyn, Poland; m.bacmaga@uwm.edu.pl (M.B.); jan.kucharski@uwm.edu.pl (J.K.)

**Keywords:** prosulfocarb, soil, contaminated, polymers, microbiota, diversity

## Abstract

Despite their effectiveness in eliminating weeds, herbicides can indirectly and directly affect organisms, leading to a decline in species abundance as well as disruptions to the structure and functioning of ecosystems. Boxer 800 EC, whose active ingredient is prosulfocarb, is an active herbicide commonly used for weed control, but its potential ecological risks are not well understood. With this in mind, a study was conducted to evaluate the effectiveness of sodium alginate and sodium polyacrylate in restoring homeostasis to soil exposed to Boxer 800 EC herbicide. This involved a two-factor pot experiment: factor I—herbicide dose (0.0, 0.8, 4.8, and 48.0 mg kg^−1^ d.m.); factor II—polymer type (soil with the polymer additives sodium alginate, and sodium polyacrylate). The experiment was carried out on Eutric Cambisols with four replicates and lasted for 50 days. The test plant was *Triticum aestivum* L., cultivar “KWS Dorium C1”. The contaminant herbicide doses inhibited the proliferation of organotrophic bacteria and actinobacteria and reduced the colony development index (CD) and ecophysiological diversity index (EP) values for these microorganisms. The addition of sodium alginate to the soil increased the proliferation of these microorganisms, whereas sodium polyacrylate inhibited their development. Sodium alginate also increased the colony development index value of organotrophic bacteria and actinobacteria. Across all the analyzed factors, bacteria from the phylum Proteobacteriota dominated. However, the presence of herbicides and polymers changed the abundance of these bacteria. Bacteria of the genus *Sphingomonas* were the most prevalent genus in the samples. The herbicide Boxer 800 EC exerted a toxic effect on the growth and development of spring wheat, which was reflected in the plant biomass yield (shoot and ear) and the SPAD index. The recommended herbicide dose (0.80 mg kg^−1^) did not cause significant changes in the growth and development of spring wheat. The hydrogel control additives deepened the negative effect of the herbicide on plant development. While the herbicide significantly reduced the levels of available carbon and total nitrogen in the soil, the polymers increased these parameters.

## 1. Introduction

Herbicides play a crucial role in effective weed control. Consequently, there is an increasing demand for the development of herbicides that are both environmentally friendly and safe for crops. In response to increasing environmental awareness and the need to minimize the negative impact of plant protection products on the natural environment, there has been a marked increase in the demand for herbicides characterized by high selectivity and limited toxicity to non-target organisms, including soil microorganisms and aquatic organisms [1]. Particular attention is being drawn to so-called “green” herbicide formulations and biopesticides, which are based on natural chemical compounds, microbial metabolites, or plant extracts and which align with the principles of sustainable agriculture [2]. These trends reflect increasingly stringent regulations on the use of synthetic pesticides and the need to reduce their residues in the environment. Therefore, it is becoming increasingly important not only to develop new active substances but also to explore methods for mitigating the negative impacts of existing herbicides on soil and cultivated plants [3]. New chemical herbicides with alternative modes provide more effective weed control, enhance agricultural productivity, and are often more environmentally friendly [4,5]. However, the long-term and improper use of herbicides with similar modes of action can lead to the development of herbicide-resistant weed populations [6,7]. Such resistance can reduce efficacy and necessitate higher doses, which can have significant environmental impacts, including increased persistence in the environment, contamination of water bodies, threats to wildlife, and potential bioaccumulation in plant, animal, and human tissues [8]. These substances can disrupt the functioning of natural and agricultural ecosystems by changing the soil microbiome and, consequently, driving evolutionary shifts [9,10,11]. Meena et al. [12] highlight the reduction in microbial populations following herbicide application, with effects dependent on the type of active ingredient used. Herbicides can influence microbial biodiversity by altering their physiology and biosynthetic pathways, which, in turn, can affect soil enzymatic activity, cell membrane composition, protein biosynthesis, and the production of plant growth regulators (such as gibberellin synthesis, indoleacetic acid (IAA) transport, and ethylene concentration, etc.). The use of excessive herbicide doses can even lead to the death of many sensitive microorganisms [13,14,15].

Prosulfocarb [s-benzyl dipropyl(thiocarbamate)] is a relatively new herbicide that belongs to the group of thiocarbamate compounds. This substance was first introduced in 1988 by Stauffer Chemical in Belgium. Currently, prosulfocarb is registered and marketed by Syngenta (Basel, Switzerland) under the trade names Boxer or Filon. In Europe, it is used for the selective control of grass and broadleaf weeds, primarily in wheat cultivation. Prosulfocarb is absorbed by both the leaves and roots of plants, leading to the inhibition of lipid biosynthesis in meristematic regions [16,17,18]. According to Braun et al. [19], this compound exhibits little or no toxicity to non-target organisms. Herbicides belonging to the thiocarbamate group contain an s-alkyl, s-benzyl or s-chlorobenzyl group and are metabolized in plants and mammals by hydroxylation and sulfoxidation to sulfoxide compounds, which can be cleaved by reactions involving enzymes and glutathione [20]. It has been shown that the sulfoxides formed during the conversion of thiocarbamates are toxic to reproduction in male rats and hepatotoxic in carp. Prosulfocarb, used in crop protection, can enter the atmosphere and be present in both gaseous and particulate phases [21]. In this study, prosulfocarb was selected due to its widespread use in agriculture and its physicochemical properties, which predispose it to extensive dispersion in the environment. Additionally, there is a lack of detailed data on its dynamics in soil environments, its stability under various environmental conditions, and its potential ecological consequences. For these reasons, prosulfocarb represents a model compound for evaluating herbicide dispersion phenomena and their secondary emissions, as well as for identifying potential environmental risks associated with the intensification of agricultural production. In addition, prosulfocarb can penetrate various environmental media such as water, soil, and sediments, making it a potential hazard to human and animal health. To mitigate the adverse effects of chemical compounds (e.g., pesticides, heavy metals, petroleum derivatives, etc.) on the soil environment, substances that immobilize these pollutants or reduce their toxicity are increasingly being used. Among these substances are hydrogels, which are frequently employed in agriculture to improve soil quality by effectively mobilizing nutrients and enhancing water retention. Their capacity to absorb and gradually release substantial amounts of water improves the soil quality and reduces the frequency of irrigation [22,23]. Incorporating hydrogels into soil enhances its structure and physical properties, such as water retention, permeability, infiltration rate, drainage, and aeration. Hydrogels are commonly used to retain water, minerals, and soil aggregates, particularly in areas prone to extreme weather events or erosion [24]. Furthermore, hydrogels positively influence plant health by improving cell membrane integrity and leaf water content, reducing xylem and phloem blockage during translocation and prolonging water availability, thus improving plant physiological parameters [22,23]. Huang et al. [25] highlight the excellent adsorption properties of hydrogels for removing pollutants. These properties arise from functional groups (e.g., carboxyl and hydroxyl) that participate in ion exchange and immobilize pollutants within the hydrogel matrix. When incorporated into soil, a hydrogel forms an amorphous gelatinous mass upon hydration and can adsorb and desorb water over an extended period [26,27,28]. These characteristics of hydrogels not only mitigate the environmental impact of agrochemicals by reducing losses through leaching, volatilization, and degradation but also maintain the efficacy of these compounds [29]. Therefore, hydrogels are considered an effective tool for supporting the proper functioning of soil ecosystems [30].

Considering the above premises, the following research hypotheses were formulated: (i) Excessive application of prosulfocarb induces negative changes in the structure and functioning of the soil microbiota and adversely affects the physiological development of spring wheat. It is hypothesized that this herbicide disrupts the microbiological balance of the soil through selective pressure on soil microorganisms, leading to reduced abundance and biodiversity by limiting the availability of nutrients. Consequently, these changes translate into oxidative stress and restricted growth of the cultivated plant. (ii) The addition of hydrogels to soil exposed to prosulfocarb contributes to the restoration of the soil’s biological and chemical homeostasis. It is assumed that hydrogels, due to their sorptive properties and ability to retain water and chemical substances, reduce the bioavailability of the herbicide to soil organisms and plant roots, thereby mitigating its toxic effects. As a result, this leads to the restoration of microbial activity, improved growth parameters of spring wheat, and stabilization of the soil’s ecosystem functions. Therefore, the aim of this study was to assess the effects of two polymers (sodium alginate and sodium polyacrylate) on the quality of soil exposed to the Boxer 800 EC herbicide and on the physiological condition of spring wheat (*Triticum aestivum* L.) grown under chemical stress. Specifically, the study aimed to (i) evaluate the extent to which the tested polymers mitigate the toxic effects of prosulfocarb on soil microbiota by analyzing changes in the abundance, diversity, and structure of prokaryotic microorganisms; (ii) determine the impact of the applied substances on the physicochemical properties of the soil, including on its pH, hydrolytic acidity, total exchangeable base cations, soil sorption capacity, degree of base saturation, and content of organic carbon and total nitrogen; and (iii) investigate the extent to which these polymers contribute to the improvement of the growth, development, and physiological parameters of spring wheat under herbicide-induced stress. The study also aimed to assess the application potential of these substances as additives supporting the restoration of homeostasis in agroecosystems disrupted by herbicides.

## 2. Results

### 2.1. Soil Microbiota

The statistical analysis (Table 1) revealed that the herbicide dose had the strongest influence on the colony development index of organotrophic bacteria (ŋ^2^ = 41.35%), while the polymer type most strongly affected the number of actinobacteria (ŋ^2^ = 96.74%), and the factor interaction of both factors had the greatest effect on the ecophysiological diversity index of organotrophic bacteria (ŋ^2^ = 74.89%).

The number of organotrophic bacteria (Org) and actinobacteria (Act) in the soil was influenced by both the herbicide dose and the type of hydrogel added to the soil (Table 2). The application of the recommended dose (0.80 mg kg^−1^ d.m. soil) stimulated microbial proliferation. Compared with the control soil (soil without herbicide addition), the number of organotrophic bacteria increased by 54.96%, while the number of actinobacteria increased by 47.13%. However, the addition of 4.8 mg kg^−1^ and 48.0 mg kg^−1^ proved toxic to the studied microorganisms: the number of organotrophic bacteria decreased by 44.27% and 53.62%, respectively, relative to the control, while the number of actinobacteria decreased by 33.88% and 31.09%, respectively. The addition of hydrogels to the soil had varying effects on the examined microbial groups. Enrichment of the soil with sodium alginate led to a 3-fold to 5.8-fold increase in the number of organotrophic bacteria and a 3.1-fold to 5.4-fold increase in actinobacteria compared with soils without hydrogel addition. In contrast, sodium polyacrylate had no significant effect on the number of microorganisms.

**Figure 1:**
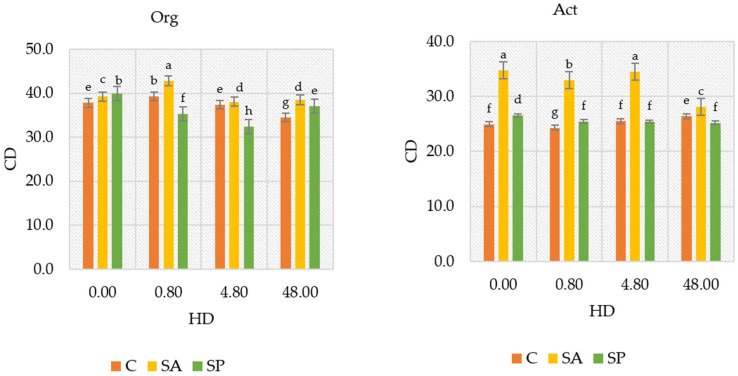
Colony development (CD) index of organotrophic bacteria and actinobacteria in soil with herbicides and polymers. The CD values range from 0 to 100. CD values close to 100 indicate a rapid growth of microbial populations within a short period. HD—herbicide dose (mg kg^−1^ d.m. soil); Org—organotrophic bacteria; Act—actinobacteria; C—control soil; SA—soil with sodium alginate; SP—soil with sodium polyacrylate;. Homogeneous groups (a–h) were calculated separately for organotrophic bacteria and actinobacteria depending on the herbicide dose and polymer type (two-way analysis of variance ANOVA performed with Tukey’s test for *p* ≤ 0.05; *n* = 4).

The colony development index (CD) reflects the growth rate of microbial colonies. Higher values may indicate the dominance of fast-growing species or stimulation of microbial metabolism. The herbicide dose of 0.80 mg kg^−1^ increased the colony development (CD) index of organotrophic bacteria from 37.781 (in the control soil) to 39.258, while the dose of 48.0 mg kg^−1^ reduced the CD index to 34.544 (Figure 1). In contrast, the CD index of actinobacteria was not significantly affected by the herbicide dose of 0.80 mg kg^−1^ and 4.80 mg kg^−1^, remaining at a similar level to the control. Only at the highest dose of 48.0 mg kg^−1^ did the CD index of actinobacteria increase from 24.951 to 26.383. In soils treated with sodium alginate or sodium polyacrylate and combined with herbicide doses ranging from 0.80 mg kg^−1^ to 48.0 mg kg^−1^, the CD index of organotrophic bacteria decreased significantly compared with the control soil. The introduction of sodium polyacrylate into the soil resulted in a decrease in the CD index of organotrophic bacteria. However, sodium alginate enhanced the CD index of both organotrophic bacteria and actinobacteria compared with treatments without the hydrogels. Sodium polyacrylate showed no significant effect on the CD index of actinobacteria.

**Figure 2:**
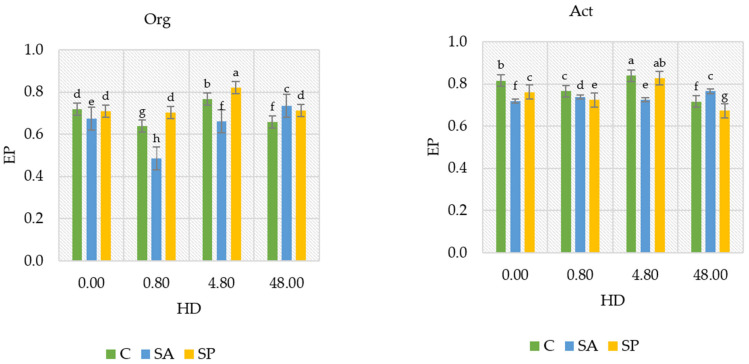
Ecophysiological diversity (EP) index of organotrophic bacteria and actinobacteria in soil with herbicides and polymers. EP values, which range from 0 to 1, indicate the stability and uniformity of microorganisms over time. EP values close to 1 indicate consistent microbial growth in the environment. HD—herbicide dose (mg kg^−1^ d.m. soil); Org—organotrophic bacteria; Act—actinobacteria; C—control soil; SA—soil with sodium alginate; SP—soil with sodium polyacrylate. Homogeneous groups (a–h) were determined separately for organotrophic bacteria and actinobacteria depending on the herbicide dose and polymer type (two-way analysis of variance ANOVA performed with Tukey’s test for *p* ≤ 0.05; *n* = 4).

The herbicide affected the ecophysiological diversity index (EP) of organotrophic bacteria and actinobacteria differently (Figure 2). The ecophysiological diversity index (EP) indicates the evenness of the contribution of different taxa within the structure. An increase in this index may reflect microbial adaptation to stress conditions or even the utilization of ecological niches. For organotrophic bacteria, the EP value decreased from 0.718 to 0.638 and 0.659 following the application doses of 0.80 mg kg^−1^ and 48.0 mg kg^−1^, respectively. For actinobacteria, the EP index decreased from 0.816 to 0.766 and 0.717 at the same herbicide doses. In soils treated with sodium alginate and a herbicide dose of 0.80 mg kg^−1^, the EP index of organotrophic bacteria decreased from 0.675 to 0.484, but at a herbicide dose of 48.0 mg kg^−1^, it increased from 0.675 to 0.736. In contrast, sodium polyacrylate in soils treated with a herbicide dose of 4.80 mg kg^−1^ increased the EP of organotrophic bacteria from 0.710 to 0.821 and that of actinobacteria from 0.761 to 0.827. However, sodium polyacrylate at a herbicide dose of 48.0 mg kg^−1^ reduced the EP index of actinobacteria from 0.761 to 0.672. Both sodium alginate and sodium polyacrylate showed contrasting effects on the EP index of organotrophic bacteria and actinobacteria compared with soils without the hydrogel additions. Sodium alginate reduced the EP index for both the groups in control soils and those treated with herbicide doses of 0.80 mg kg^−1^ to 4.8 mg kg^−1^, while it increased the EP index at the highest herbicide dose (48.0 mg kg^−1^). Sodium polyacrylate, on the other hand, tended to increase the EP index of organotrophic bacteria and actinobacteria at herbicide doses of 0.80 mg kg^−1^ to 4.80 mg kg^−1^ but had a reducing effect at 48.0 mg kg^−1^.

**Figure 3:**
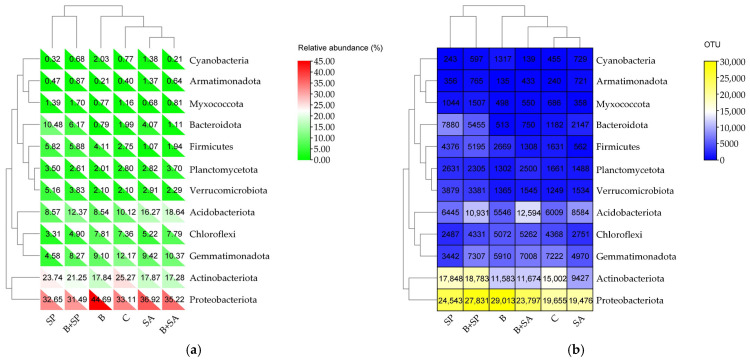
Dominant phylum of bacteria in the soils: (**a**) the relative abundance of bacteria and (**b**) the number of OTUs of bacteria (OTU ≥ 1%). C—control soil; B—soil contaminated with the Boxer 800 EC herbicide; SA—soil supplemented with sodium alginate; B + SA—soil contaminated with herbicide and supplemented with sodium alginate; SP—soil supplemented with sodium polyacrylate; B + SP—soil contaminated with herbicide and supplemented with sodium polyacrylate.

Under the influence of the Boxer 800 EC herbicide and hydrogels, significant changes were observed in the structure of bacterial communities at the phylum level (Figure 3). Bacteria belonging to the phylum Proteobacteriota dominated in all the soil samples, comprising 31.49% to 44.69% of the total bacterial population. Actinobacteriota were also abundant (17.28% to 25.27%), followed by Acidobacteriota (8.54% to 18.64%), Gemmatimonadota (4.58% to 12.17%), and Chloroflexi (3.31% to 7.81%). At site B, the number of Proteobacteriota OTUs increased by 47.61% compared with site C (control soil), while at the B + SA, B + SP, and SP sites, increases of 22.19%, 13.40%, and 3.13%, respectively, were recorded. However, at site SA, the number of Proteobacteriota OTUs decreased by 32.87%. The herbicide treatment decreased the number of OTUs belonging to the phyla Acidobacteriota, Actinobacteriota, Armatimonadota, Bacteroidota, Gemmatimonadota, Myxococcota, and Planctomycetota. In contrast, it increased the number of OTUs of Chloroflexi, Cyanobacteria, Firmicutes, and Verrucomicrobiota. In soils amended solely with sodium alginate or sodium polyacrylate, the number of OTUs of Acidobacteriota, Armatimonadota, Bacteroidota, and Verrucomicrobiota increased, while that of Chloroflexi and Gemmatimonadota decreased. In soils treated with both hydrogels and herbicides, the number of OTUs of Acidobacteriota, Actinobacteriota, Chloroflexi, Firmicutes, Gemmatimonadota, Myxococcota, and Proteobacteriota increased compared with the control soil (soil without the addition of herbicides and hydrogels).

**Figure 4:**
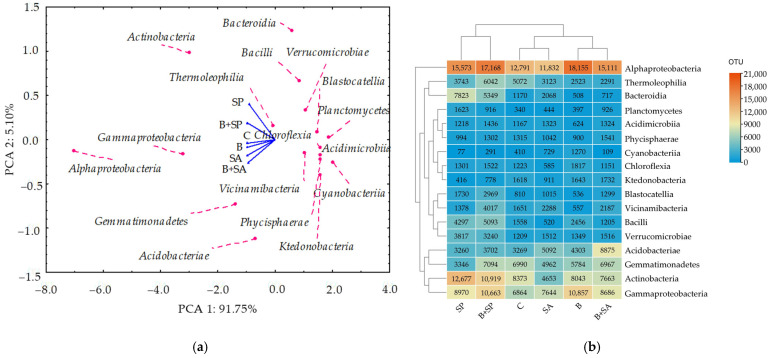
Dominant classes of bacteria in the soils (OTU ≥ 1%) presented as (**a**) PCA (Principal Component Analysis), and (**b**) heat map. C—control soil; B—soil contaminated with the Boxer 800 EC herbicide; SA—soil supplemented with sodium alginate; B + SA—soil contaminated with herbicide and supplemented with sodium alginate; SP—soil supplemented with sodium polyacrylate; B + SP—soil contaminated with herbicide and supplemented with sodium polyacrylate.

Principal component analysis (PCA) revealed that PCA 1 accounted for 91.75% of the total variance, while PCA 2 explained 5.10% (Figure 4). The most abundant bacterial class across all the studied sites was *Alphaproteobacteria*, with OTU counts ranging from 11,832 to 18,155. This was followed by *Gammaproteobacteria* (from 6864 to 10,857 OTUs), *Actinobacteria* (from 4653 to 12,677 OTUs), *Acidobacteriae* (from 3260 to 8875 OTUs), and *Thermoleophilia* (from 2291 to 6042). The analysis showed an increase in *Alphaproteobacteria* in soils B, B + SA, SP, and B + SP compared with control soil C; in *Gammaproteobacteria* in soils B, SA, B + SA, SP, and B + SP; in *Actinobacteria* in soils SP and B + SP; in *Acidobacteriace* in soils B, SA, and B + SA; and in *Thermoleophilia* in soils B + SP. PCA demonstrated that *Alphaproteobacteria* were the furthest from the vectors, indicating the highest abundance of these bacteria in the soil samples. Conversely, *Thermoleophilia* were closest to the vectors, indicating the least divergence in their numbers among the samples. The PCA plot formed four distinct clusters. The first quadrant contained a unicellular group comprising *Bacilli*, *Bacteroidia*, *Blastocatellia*, *Planctomycetes* and *Thermoleophilia*. The second quadrant included bacteria from the class *Actinobacteria*. The third quadrant grouped *Alphaproteobacteria*, *Gammaproteobacteria*, *Acidobacteria,* and *Gemmatimonadetes*; while the fourth quadrant comprised *Acidimicrobiia*, *Cyanobacteria*, *Ktedonobacteria*, *Phycisphareae,* and *Vicinamibacteria*.

**Figure 5:**
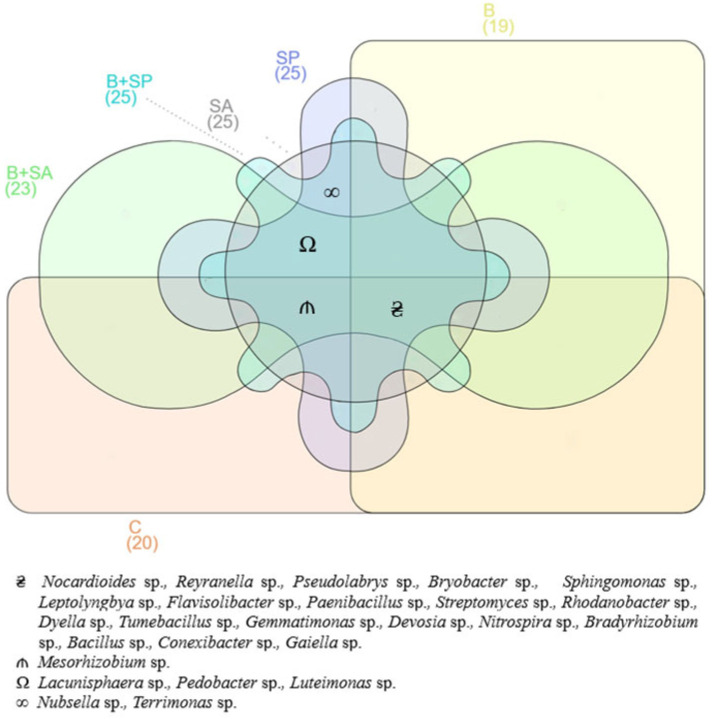
Unique and common genera of bacteria in the soils (OTU ≥ 1%). C—control soil; B—soil contaminated with the Boxer 800 EC herbicide; SA—soil supplemented with sodium alginate; B + SA—soil contaminated with herbicide and supplemented with sodium alginate; SP—soil supplemented with sodium polyacrylate; B + SP—soil contaminated with herbicide and supplemented with sodium polyacrylate.

The Venn diagram (Figure 5) shows that all soils examined were colonized by 19 of the 25 identified bacterial genera: *Nocardioides, Reyranella, Pseudolabrys, Bryobacter, Sphingomonas, Leptolyngbya, Flavisolibacter, Paenibacillus, Streptomyces, Rhodanobacter, Dyllea, Tumebacillus, Gemmatimonas, Devosia, Nitrosospira, Bradyrhizobium, Bacillus, Conexibacter*, and *Gaiella*. Bacteria of the genus *Mesorhizobium* appeared exclusively in soils SP and B + SP. In soil SA, SP, and B + SP, the genera *Nubsella* and *Terrimonas* were observed. In soils SA, B + SA, and SP, the genera *Lacunisphaera, Pedobacter,* and *Luteimonas* were present.

**Figure 6:**
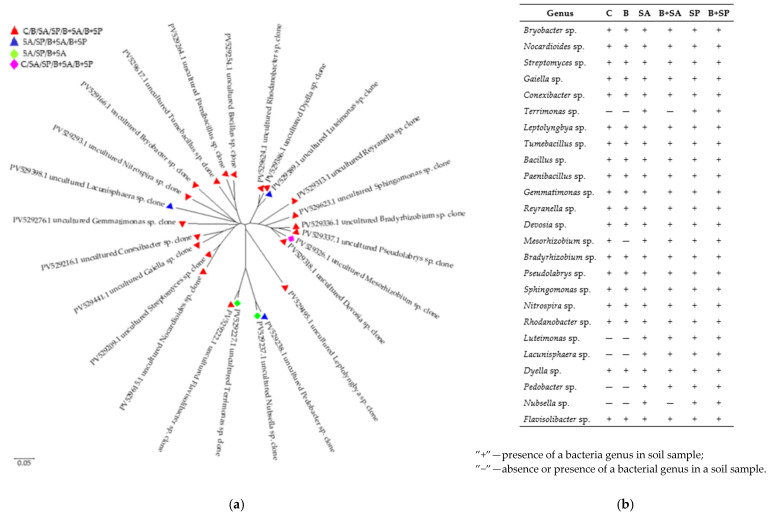
Dominant genera of bacteria in the soil shown as (**a**) the phylogenetic tree of bacteria isolated from soils contaminated with herbicides and enriched with sodium alginate and sodium polyacrylate was constructed using MEGA 7 software by the neighbor-joining (NJ) method based on 16S rRNA gene sequences, and (**b**) genus of bacteria identified in soil samples. C—control soil; B—soil contaminated with the Boxer 800 EC herbicide; SA—soil supplemented with sodium alginate; B + SA—soil contaminated with herbicide and supplemented with sodium alginate; SP—soil supplemented with sodium polyacrylate; B + SP—soil contaminated with herbicide and supplemented with sodium polyacrylate.

In all the soil samples (Figure 6), the most common bacterial genera were *Sphingomonas, Gemmatimonas,* and *Bryobacter*. *Streptomyces* were abundant in the C, SP, and B + SP soil samples; *Tumebacillus, Paenibacillus, Pedobacter*, and *Flavisolibacter* were abundant in the SP and B + SP soils; *Pseudolabrys* was most abundant in the B and B + SA soils; and *Nocardioides* was present in the C and B soils. The phylogenetic tree illustrating the genetic relationships between the bacterial genera identified in the soils, based on 16S rRNA gene nucleotide sequences, revealed four distinct groups (Figure 6a). The first group in terms of genetic similarity includes bacteria of the genera *Rhodanobacter, Dyella, Luteimonas, Reyranella, Sphingomonas, Bradyrhizobium, Mesorhizobium, Devosia,* and *Pseudolabrys*. The second group consists of bacteria of the genera *Leptolyngbya, Nubsella, Pedobacter, Flavisolibacter*, and *Terrimonas*. The third group with high genetic homology consists of bacteria belonging to the genera *Bryobacter, Nitrospira, Lacunisphaera, Gemmatimonas, Conexibacter, Gaiella, Streptomyces* and *Nocardioides*. The fourth group is represented by the genera *Paenibacillus, Bacillus*, and *Tumebacillus*. It was found that 19 bacterial genera were present in all the soil samples (Figure 6b). Additionally, *Luteimonas, Lacunisphaera*, and *Pedobacter* were absent from soils C and B; *Terrimonas* and *Nubsella* were absent from soils C, B, and B + SA; and *Mesorhizobium* was absent from soil B.

**Figure 7:**
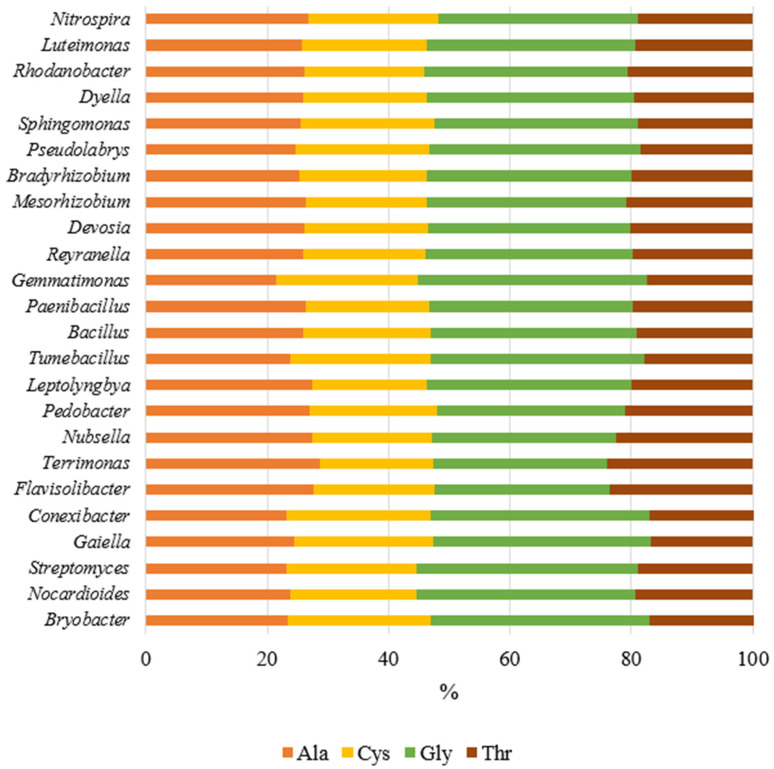
Predicted amino acid composition calculated from bacterial nucleotide sequences using the ProtParam database on ExPasy [31,32]. Ala—alanine; Cys—cysteine; Gly—glycine; Thr—threonine.

Based on the nucleotide sequences of the isolated bacterial genera from the soil, the predicted physicochemical properties of the peptides (Table 3) and their amino acid composition (Figure 7) were determined.

The molecular weight range of the peptides ranged from 31.625 kDa (*Reyranella* sp.) to 33.968 kDa (*Flavisolibacter* sp.). Peptides from the 27 bacterial genera examined had an isoelectric point (pI) value ranging from 5.07 to 5.09, indicating that these were acidic proteins (Table 3). The aliphatic index, reflecting the relative volume occupied by the side chains of amino acids such as alanine, valine, leucine, and isoleucine, was highest for *Terrimonas* (28.67) and lowest for *Gemmatimonas* (21.28). This confirms the presence of alanine and similar amino acids in the peptide composition. The molar extinction coefficient (ExC) values ranged from 4875 to 6250, suggesting variations in the content of amino acids like cysteine that absorb light at 280 nm. The instability index, which indicates protein stability, revealed that the most stable proteins were found in *Streptomyces*, *Nubsella*, *Bacillus*, *Paenibacillus*, *Mesorhizobium*, *Rhodanobacter*, and *Luteimonas*, as evidenced by an index value of below 40.0. In our studies, the aliphaticity index value was between 21.28% and 28.67%, confirming the presence of alanine in the amino acid composition. The hydropathicity index (GRAVY), reflecting the solubility of the peptides, was positive for all the bacterial genera studied, indicating the hydrophobic nature of the proteins.

The percentage of amino acids was calculated for all bacterial genera accounting for more than 1% of the OTU (Figure 7). The analysis revealed that four amino acids predominated in the sequences: alanine, cysteine, glycine, and threonine. Alanine and threonine were found in the highest amounts in the peptides from *Terrimonas,* accounting for 28.70% and 23.90% of the total amino acid composition, respectively. Cysteine dominated the peptides of *Bryobacter* and *Conexibacter*, making up 23.70% of the amino acid composition. Glycine was most abundant in the peptides of *Gemmatimonas*, accounting for 37.70% of the total amino acid composition.

**Figure 8:**
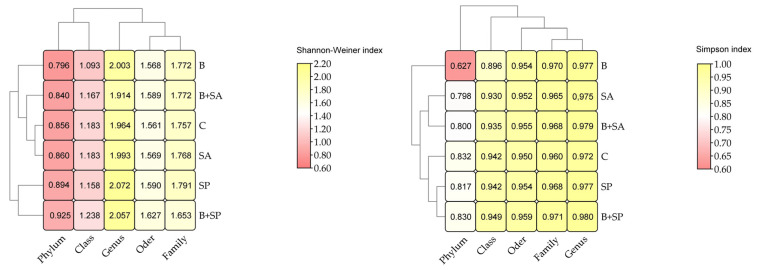
Shannon–Wiener index and Simpson index in the soils. C—control soil; B—soil contaminated with the Boxer 800 EC herbicide; SA—soil supplemented with sodium alginate; B + SA—soil contaminated with herbicide and supplemented with sodium alginate; SP—soil supplemented with sodium polyacrylate; B + SP—soil contaminated with herbicide and supplemented with sodium polyacrylate.

The changes that occurred in the genetic diversity of the bacteria were also reflected in the values of the Shannon–Wiener index and the Simpson index (Figure 8). In soil B, the values of the Shannon–Wiener index and the Simpson index decreased compared with those in soil C at the strain and class level. In soils SA and SP, the Shannon–Wiener index increased at the stem, order, family and genus levels; in soil B + SA, it increased at the order and family level; while in soil B + SP, it increased at the stem, class, order, and genus levels. For the Simpson index, a decrease in value was observed in soils SA and B + SA at the stem and class level and in soil SP at the stem level. In soil B + SA, the Simpson index increased from the class to the genus level.

### 2.2. Growth and Development of Spring Wheat

The herbicide dose (Table 4) had the greatest effect on the growth and development of spring wheat, with values ranging from 82.05% (PDM) to 99.35% (PEL). The type of polymer had little effect on the growth and development of spring wheat, as the ŋ^2^ value ranged from 0.01% (LGI) to 7.50% (PDM).

The application of herbicides to the soil resulted in significant changes in the growth and development of *Triticum aestivum* L. (Table 5 and Figure S1). The highest herbicide dose of 48.0 mg kg^−1^ d.m. of soil had a toxic effect on spring wheat, resulting in complete plant loss due to seedling damage. Compared with the control sample, the dose of 4.80 mg kg^−1^ decreased the dry matter yield of the plants by 15.99%, reduced the length of the aerial parts by 5.68%, and lowered the SPAD index by 13.12%, while the ear length was increased by 3.13%. The addition of sodium alginate to the soil significantly inhibited the growth and development of spring wheat compared with the soil without hydrogels. The plant dry matter yield decreased by 29.49% (soil without herbicide) to 69.92% (soil with a herbicide dose of 4.80 mg kg^−1^), the length of the aerial parts decreased by 6.44% (soil without herbicide) to 22.09% (soil with a herbicide dose of 4.80 mg kg^−1^), and the ear length decreased by 1.54% (soil without herbicide) to 6.06% (soil with a herbicide dose of 4.80 mg kg^−1^). In plots with sodium alginate, the SPAD index decreased by 5.80 in soils containing 0.80 mg kg^−1^ of herbicide, while it increased by 11.19% at a dose of 4.80 mg kg^−1^. Sodium polyacrylate also had a negative effect on *Triticum aestivum* L. The dry matter yield of the plants decreased by 2.11% (soil with a herbicide dose of 4.80 mg kg^−1^) to 34.05% (soil without herbicide), while the length of the aerial parts decreased by 2.70% (soil with a herbicide dose of 0.80 mg kg^−1^) to 16.49% (soil with a herbicide dose of 4.80 mg kg^−1^).

### 2.3. Physicochemical Properties of the Soil

It was found that the herbicide dose had the greatest effect on the total nitrogen content, while polymer type had the most significant impact on the pH, soil hydrolytic acidity, sum of basic exchangeable cations, soil sorption capacity, and degree of soil saturation with basic cations. The interaction between these factors influenced the organic carbon content and the ratio of organic carbon content to total nitrogen content (Table 6).

Boxer 800 EC applied to the soil at of 0.80 mg kg^−1^ and 48.0 mg kg^−1^ doses reduced the organic carbon content (C_org_) by 4.82% and 5.56% and the total nitrogen content (N_tot_) by 12.84% and 5.36% at 0.80 mg kg^−1^ and 4.80 mg kg^−1^, and the carbon to nitrogen ratio (C/N) by 4.62% at 48.0 mg kg^−1^, respectively, compared with a soil without herbicide. It did not significantly affect the other physicochemical properties of the soil (pH, HAC, EBC, CEC, and BS) (Table 7). Supplementation of the soil with sodium alginate, compared with soil without polymers, increased the C_org_ by 5.70 and 19.81 and the N_tot_ by 6.60 and 6.67 at sites with herbicide application rates of 4.0 mg kg^−1^ and 48.0 mg kg^−1^, respectively. Sodium polyacrylate increased the levels of C_org_ and N_tot_ in the control soil (by 33.87% and 11.87%, respectively) and in soil treated with herbicide at 0.80 mg kg^−1^ (by 20.73% and 7.08%, respectively), while it decreased these levels in the soil with the highest herbicide dose (48.0 mg kg^−1^) by 18.46% and 7.14%, respectively. In soils without herbicide and with herbicide at doses ranging from 0.80 mg kg^−1^ to 48.0 mg kg^−1^, both sodium alginate and sodium polyacrylate increased the value of total exchangeable base cations (EBC) and soil saturation with base cations (CEC).

### 2.4. Correlations Between Soil Microbial Activity, Soil Physicochemical Properties, and Spring Wheat Growth and Development

**Figure 9:**
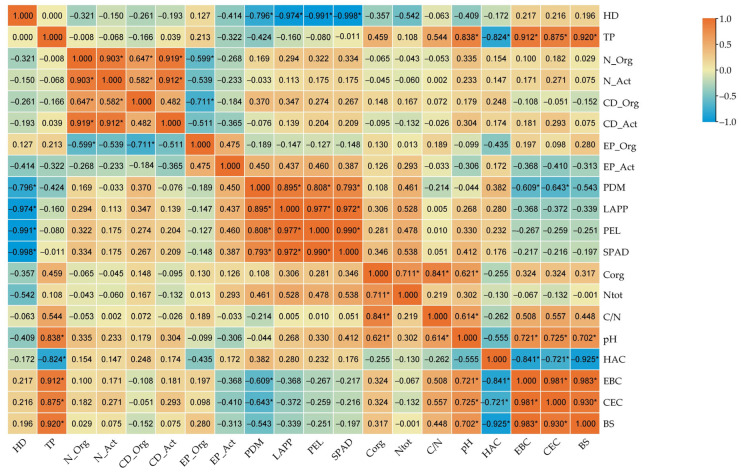
Simple Pearson’s correlation coefficients between the analyzed parameters (*n* = 12; *p* ≤ 0.05). HD—herbicide dose; TP—type of polymer; N_Org—numbers of organotrophic bacteria; N_Act—numbers of actinobacteria; CD_Org—organotrophic bacteria colony development index; CD_Act—actinobacteria colony development index; EP_Org—ecophysiological diversity index of organotrophic bacteria; EP_Act—ecophysiological diversity index of actinobacteria; PDM—plant dry matter; LAPP—length of above-ground parts of plants; PEL—plant ear length; SPAD—SPAD index; C_org_—organic carbon content; N_tot_—nitrogen carbon content; C/N—ratio of organic carbon content to total nitrogen content; pH—soil pH in 1 M KCl dm^−3^; HAC—hydrolytic acidity; EBC—sum of exchangeable bases; CEC—sorption capacity; BS—base saturation; *—statistically significant at *p* ≤ 0.05.

The correlation analysis between the investigated parameters (Figure 9) showed significant negative correlations between herbicide dose and the following: PDM (plant dry mass), LAPP (length of above-ground plant parts), PEL (plant ear length), and SPAD index (leaf green index); polymer type and soil hydrolytic acidity; organotrophic bacterial number and organotrophic bacterial growth index vs. the ecophysiological diversity index of organotrophic bacteria; plant dry matter yield vs. total exchangeable alkaline cations and soil sorption capacity; hydrolytic acidity vs. total exchangeable alkaline cations, soil sorption capacity, and degree of soil saturation with base cations. Significant positive correlations were observed between polymer type and soil pH, the sum of exchangeable cations after plant emergence, sorption capacity, and soil saturation with base cations; the number of organotrophic bacteria and actinobacteria; the colony development index of organotrophic bacteria and that of actinobacteria; the number of actinobacteria and their colony development index; plant dry matter yield and the length of the above-ground parts, spike length, and the SPAD index; the length of the above-ground parts and spike length; plant ear length and the SPAD index; organic carbon content and total nitrogen content, C/N ratio, and pH; pH and the sum of exchangeable alkaline cations, soil sorption capacity, and degree of saturation with base cations; the sum of exchangeable alkaline cations and soil sorption capacity and the degree of saturation of with base cations; and soil sorption capacity and the degree of saturation with base cations.

## 3. Discussion

### 3.1. Soil Microbiota

The uncontrolled use of herbicides can lead to changes in the biodiversity of the soil ecosystem. The effects of some herbicides on soil microbiota have not yet been fully investigated. An example of such a herbicide is Boxer 800 EC (whose active ingredient is prosulfocarb), which, in the present study, contributed to the inhibition of the proliferation of organotrophic bacteria and actinobacteria. This effect was particularly evident following the application of the highest dose of 48.0 mg kg^−1^. The number of organotrophic bacteria decreased by 53.62% compared with the control sample, while the number of actinobacteria decreased by 31.09%. However, this herbicide contributed to an increase in the colony development index (CD) for actinobacteria, which may result from the selective effect of the herbicide in promoting more tolerant and faster-growing strains. Barba et al. [33] observed that prosulfocarb promoted the growth of Gram-negative bacteria while reducing the growth of Gram-positive bacteria. A reduction in the number of Gram-positive bacteria was also observed following the application of other herbicides such as chlorotoluron and a mixture of flufenacet and diflufenican, which can be attributed to the greater sensitivity of Gram-positive bacteria to environmental stress compared with Gram-negative bacteria [34].

Changes in the structure of bacterial communities were also observed in this study following the application of the herbicide Boxer 800 EC. Soil contaminated with this herbicide was dominated by bacteria belonging to the phylum Proteobacteriota, whose relative abundance increased by 47.61% compared with the control soil. Wang et al. [35] also reported the highest relative abundance of Proteobacteriota in soil contaminated with chlorosulfuron-methyl. Proteobacteriota represent a ubiquitous bacterial phylum in soils due to their wide range of functions, particularly their involvement in the cycling of key elements such as nitrogen, carbon, and sulfur. The relative abundance of these bacteria increases as the availability of organic carbon in the soil rises [36]. Proteobacteriota may use herbicides as a carbon or energy source, leading to the degradation of these pollutants to harmless or less toxic compounds and, thereby, reducing the herbicide content in the soil [37]. Actinobacteriota, Acidobacteriota, and Gemmatimonadota were also found to be quite abundant in the soil samples analyzed in our study. Laboratory experiments with prosulfocarb conducted by Barba et al. [33] indicated a predominance of Actinobacteriota, although their relative abundance decreased in soil containing 10 mg kg^−1^ prosulfocarb compared with control soils. Similar correlations were observed in our study, where the relative abundance of Actinobacteriota decreased by 28.97% compared with control soils. Carpio et al. [34], reported that herbicides tend to promote the growth of Actinobacteriota, as they are among the most widespread and abundant bacterial phylotypes in soil. Consequently, they are highly versatile in colonizing a wide range of environments and may exhibit resistance to xenobiotics, including herbicides. According to Du et al. [38] and Onyango et al. [39], another group of bacteria capable of degrading organic material into simpler compounds is Gemmatimonadota. However, despite these capabilities, these bacteria could not withstand the stress induced by prosulfocarb in our study, as their relative abundance decreased significantly. Petrić et al. [40] emphasize that microbiological adaptation is a phenomenon that enables bacteria to tolerate exposure to toxic substances. Physiological acclimatization or genetic inheritance facilitates the selection of populations that more tolerant and better able to cope with toxic chemicals. *Alphaproteobacteria* were the most numerous of the identified bacterial classes, followed by slightly fewer *Actinobacteria* and *Gammaproteobacteria*. These classes play crucial roles in the soil environment. Bacteria belonging to the *Alphaproteobacteria* and *Gammaproteobacteria* classes, are involved in the transformation of inorganic compounds and nitrogen fixation. They can degrade, biosynthesize and utilize a wide range of compounds in the soil and exhibit high resistance to temperature fluctuations and nutrient deficiencies [41]. In addition, *Alphaproteobacteria* can establish relationships with plant roots, enhancing nutrient availability and promoting plant growth and development [42]. *Actinobacteria* contribute to the cycling of organic compounds and the formation of soil organic matter through the production of melanin in association with humic acids in the soil [43]. *Sphingomonas*, a genus belonging to the class *Alphaproteobacteria*, was the dominant bacterial genus, although its abundance decreased by 36.28% compared with the control sample. This genus is widespread in the environment and is characterized by diverse metabolic capabilities. Some species within this genus are capable of removing organic pollutants [44,45,46]. In our study, *Sphingomonas* showed reduced resistance to the tested herbicide, as confirmed by the calculated protein instability index, which exceeded 40 [47]. Bacterial biodiversity, assessed using the Shannon–Wiener index and the Simpson index, also changed under the influence of the herbicide. The results demonstrated a decrease in the values of these indices at both the phylum and class levels.

Soil bacteria in the rhizosphere play a crucial role in the nutrient exchange between soil and plants and, therefore, are of great importance in soil ecosystems. The biodiversity of bacteria in soils exposed to organic pollutants can be enhanced by introducing various substances that stimulate bacterial development. Substances such as biochar [48], alginate, sepiolite, halloysite, molecular sieves [49,50], and hydrogels [51] can modify the structure of bacterial communities and increase their diversity. In our study, we used sodium alginate and sodium polyacrylate to mitigate the negative effects of the herbicide on soil microbiota. The addition of sodium alginate to the soil increased the number of organotrophic bacteria and actinobacteria. Baćmaga et al. [51] also observed stimulation of the proliferation of organotrophic bacteria and actinobacteria following the addition of sodium alginate to soils exposed to sulcotrione. The positive effect of sodium alginate on soil microbiota may be attributed to its chemical structure (specifically, the presence of carboxyl and hydroxyl groups) and its potential to adsorb pollutants from the soil environment [52,53,54,55]. In contrast, the addition of sodium polyacrylate was found to enhance the negative effect of the herbicide. This effect could be related to the polymer’s low molecular weight and long-chain structure, which may influence its interaction with environmental factors [56]. In their study, Yakimenko et al. [57] reported a stimulating effect of lignite and lignosulfonate on the activity of heterotrophic aerobic bacteria, evidenced by increased respiration and microbial biomass. In our study, supplementation of the soil with sodium alginate and sodium polyacrylate, in both herbicide-free and herbicide-contaminated soils, significantly reduced the relative abundance of Actinobacteriota and Gemmatimonadota. Conversely, the relative abundance of Acidobacteriota increased significantly in soil with sodium alginate, in both herbicide-contaminated and herbicide-free soil conditions, as well as in soils treated with sodium polyacrylate and contaminated with herbicide. Wang et al. [58] also reported that sodium polyacrylate reduced the relative abundance of Actinobacteriota, Bacteroidetes, and Firmicutes. Adjuik et al. [59] observed that sodium polyacrylate degrades more slowly in soil compared with other polymers due to its long-chain molecular geometry, which limits the availability of reactive end groups for certain bacterial enzyme systems. In our study, prosulfocarb adsorbed by sodium polyacrylate became inaccessible to microorganisms capable of its degradation, which may have been detrimental to bacterial populations sensitive to the tested herbicide. Natural hydrogels such as sodium alginate are more susceptible to degradation due to the presence of diverse carbon chains in their backbone structure. Rapid decomposition of this polymer in the soil could result in the breakdown of all organic components within its structure, leading to the release of carbon into their biomass [60].

Our study revealed that at least 19 out of the 25 identified bacterial species were present in all the analyzed soil samples. These included bacteria from the genera *Nocardioides*, *Reyranella*, *Pseudolabrys*, *Bryobacter*, *Sphingomonas*, *Leptolyngbya*, *Flavisolibacter*, *Paenibacillus*, *Streptomyces*, *Rhodanobacter*, *Dyella*, *Tumebacillus*, *Gemmatimonas*, *Devosia*, *Nitrospira*, *Bradyrhizobium*, *Bacillus*, *Conexibacter*, and *Gaiella*. These bacteria exhibited resilience to environmental changes induced by both herbicide application and hydrogel treatments. Their adaptation to altered environmental conditions is likely linked to the physicochemical properties of the proteins. The proteins produced by these bacteria contain hydrophobic amino acid residues arranged in specific patterns within their polypeptide chains. These structural characteristics determine the surface properties of the proteins, their capacity to form oligomers and micellar structures, and their functional roles. All the identified bacterial species have proteins with hydrophobic amino acid residues, whose specific arrangement in polypeptide chains characterizes the surface of their molecules, their ability to form oligomers and micellar structures, as well as their functional properties. This, in turn, enhances the stability of these bacterial species in challenging environmental conditions [61,62].

### 3.2. Growth and Development of Spring Wheat

Prosulfocarb is an effective herbicide primarily used in winter cereals to control a broad spectrum of broadleaf and grassy weeds. It inhibits key metabolic processes, including fatty acid and lipid biosynthesis, protein synthesis, isoprenoid and flavonoid biosynthesis, gibberellin production, and photosynthesis, which is why it is classified as a “multi-site” herbicide [16]. In the present study, Boxer 800 EC (active ingredient: prosulfocarb) was applied at contaminating doses and found to be toxic to spring wheat. The highest dose tested (48.0 mg kg^−1^) caused complete damage to seedlings at the beginning of the experiment. Even a lower dose of 4.8 mg kg^−1^ led to a significant reduction in spring wheat yield, along with decreases in the above-ground biomass, ear length, and SPAD index. This phytotoxic effect is likely due to the herbicide’s uptake by plant roots, resulting in severe inhibition of growth at higher doses. A secondary mechanism of prosulfocarb involves uptake through both the coleoptile and the roots, leading to suppressed shoot, root, and leaf growth. Plants absorbing the herbicide near the coleoptile node are particularly sensitive to its toxic effects [63]. Additionally, prosulfocarb-induced oxidative stress may elevate reactive oxygen species levels in plant tissues, causing damage to DNA, proteins, lipids, and other vital biomolecules. This damage could ultimately impair plant growth and development, resulting in yield losses [64]. During herbicide-induced stress, levels of malondialdehyde—a byproduct of lipid peroxidation—increase in plant shoots [65]. This accumulation may disrupt the photosynthetic process, as indicated by the 13.12-fold reduction in the SPAD index observed with a 4.80 mg kg^−1^ dose compared with the control. Similar disturbances have been reported in rice treated with butachlor, where reduced biomass, shoot length, and chlorophyll content were observed [45]. Alachlor, applied at concentrations of 1.00 to 8.00 mg dm^–3^, similarly inhibited maize shoot growth by 30.70% to 55.10% [66].

Hydrogels play a crucial role in modern agriculture, especially in the sustainable management of water resources and nutrients. Due to their chemical structure, which is rich in hydrophilic groups and characterized by a high degree of cross-linking, hydrogels can absorb and store large amounts of water and nutrients, thereby increasing their availability to plants during periods of water stress or nutrient deficiency. Moreover, the use of hydrogels can reduce water loss in soil and improve fertilizer use efficiency, contributing to decreased environmental pollution [67,68]. The enrichment of the soil with polymers intensified the negative effect of the herbicide on spring wheat. Even in soils not treated with herbicides, the presence of polymers inhibited plant growth and development. This plant response may be attributed to the hydrogel’s capacity to retain nutrients, thereby rendering them unavailable to the plants and causing disruption to their growth and development. Hydrogels, due to their numerous hydrophilic groups and high cross-linking density, are capable of swelling and absorbing water and dissolved substances, effectively immobilizing them and limiting their availability to plants [24,69]. Specifically, sodium alginate is characterized by poor stability in the environment and susceptibility to desiccation, which could lead to insufficient water availability for plant development and growth [70]. The decline in the condition of spring wheat may also be associated with changes in the cross-linking density of hydrogels and alterations in the cation concentration. These changes can limit the availability of essential cations to plants, resulting in nutrient or salt stress that impairs plant health and productivity [71].

In summary, the results obtained indicate that the use of prosulfocarb adversely affects the growth and development of spring wheat, disrupting the plants’ physiological balance and, thus, reducing the dry matter yield. Within the scope of the present study, although polymers such as sodium alginate and sodium polyacrylate may, under certain conditions, limit plant development (e.g., through excessive nutrient binding or destabilization of the soil structure), when appropriately selected and applied, they can constitute an important component of sustainable agriculture strategies and the reclamation of degraded soils. In the longer term, the development and optimization of hydrogel applications may support agricultural production while reducing the environmental pressure resulting from intensive agrochemical use [67,68].

### 3.3. Physicochemical Properties of Soil

The herbicide Boxer 800 EC contributed to modifications in the physicochemical properties of the soil, with its effect depending on the applied dose. In general, it caused a decrease in the organic carbon and total nitrogen content and an increase in the carbon-to-nitrogen ratio. Ataikiru et al. [72] report that the content of herbicides in the soil depends on the amount of organic carbon. The higher the organic carbon content in the soil, the lower the concentration of herbicides, likely due to the higher microbial activity. Omar and Tasi’u [73] observed a decrease in soil organic carbon content when atrazine and paraquat were used individually and in combination in maize cultivation. Tudararo-Aherobo and Ataikiru [74] found no significant changes in soil organic carbon and nitrogen content under the influence of atrazine and glyphosate. However, the soil pH increased significantly after the application of these herbicides compared with the control soil. In soils with a higher pH, a lower amount of herbicides binds to soil particles, making them more available to plants, which negatively affects plant health. In our study, the soil pH decreased slightly after herbicide application compared with the control soil, while hydrolytic acidity, the sum of basic exchangeable cations, and the sorption capacity of the soil increased. Fasinmirin [75] emphasises that the increase in sorption capacity could be related to the high organic matter content on the soil surface, which facilitates cation exchange. Changes in the physicochemical properties of the soil under the influence of the herbicide could also be related to the diversity of soil-inhabiting bacteria. Through their activity, soil microbiota may alter the physicochemical properties of the soil and the organic matter content [76]. Additionally, the types of bacteria isolated from the soil may have contributed to herbicide inactivation by developing defense mechanisms, including proteins, that rendered them resistant to excessive amounts of the herbicide. Moreover, the proteins presumably in bacteria from the genera *Streptomyces, Nubsella, Bacillus, Paenibacillus, Mesorhizobium, Luteimonas*, and *Rhodanobacter* had an instability index value of below 40. These predicted protein properties indicate their stability in an environment exposed to stressful conditions [77,78]. In this study, the incorporation of polymers into the soil improved its physicochemical properties. Both sodium alginate and sodium polyacrylate contributed to reducing the negative effects of prosulfocarb on soil properties. The positive effect of hydrogels on the soil physicochemical parameters is due to their stable, cross-linked 3D structure, which provides a high adsorption capacity for water and various substances. They are also characterized by the ability to change their chemical structure and volume in response to environmental changes, e.g., in pH, temperature, salt concentration, etc. Their unique physical properties, including porosity and swelling, make them suitable platforms for the controlled release of water and nutrients [79]. In addition, hydrogels can reduce the bulk density of the soil by creating a larger pore space during the drying process of swelling [80]. Erci et al. [81] define hydrogels as soil stabilizers, as they can form networks that serve as bridges between the soil surface and its particles, leading to increased soil aggregation. Studies by Abrishman et al. [82] and El-Saied et al. [83] indicate that the addition of hydrogels to soil increases its sorption capacity, which may be related to the availability of exchange sites, depending on the soil pH.

## 4. Materials and Methods

### 4.1. Soil Material

The soil used for the study originated from an agricultural area in the northeastern part of Poland (53.713° N, 20.432° E). The soil was collected from the arable humus horizon at a depth of 0–20 cm. It was classified as Eutric Cambisols soil [84] and consisted of sandy loam (sand content—52.19%; silt content—0.62%; clay content—49.17%). The pH of the tested soil in 1 mol KCl dm^−3^ was 4.73, the hydrolytic acidity (HAC) was15.190 mmol^+^ kg^−1^, the total exchangeable base cations (EBC) amounted to 28.00 mmol^+^ kg^−1^, the soil sorption capacity (CEC)was 43.19 mmol^+^ kg^−1^, the soil saturation with basic cations (BS) was 64.83%, the total nitrogen content (N_tot_) was 1.05 g kg^−1^, the organic carbon content (C_org_) was 7.05 g kg^−1^_,_ and the carbon-to-nitrogen ratio (C/N) was 6.71.

### 4.2. Herbicide Applications

The herbicide Boxer 800 EC, which contains an active substance called prosulfocarb (800 g dm^−3^ of the preparation), was used for the tests. The properties of the active substance are listed in Table 8. Syngenta Polska (Warsaw, Poland) received approval for the introduction of this preparation on the Polish market in 2015 (No. R–88/2015, approved by the Ministry of Agriculture and Rural Development on 29 May 2015). Boxer 800 EC is available in the form of a concentrate for the production of an aqueous emulsion. It can be applied both in the soil and to foliage. It is intended for the selective control of monocotyledonous and dicotyledonous weeds in agricultural and horticultural crops. The dose recommended by the manufacturer for the protection of cereals (e.g., wheat, triticale, rye, barley, millet) is 3.0 dm^3^ ha^−1^, 4.0–5.0 dm^3^ ha^−1^ for vegetables (e.g., potatoes, onions, leeks, carrots, parsley, garlic) and 3.0–4.0 dm^3^ ha^−1^for legumes (e.g., field beans, field peas, narrow-leaved lupin, yellow lupin, white lupin, lentils).

Weeds show the following responses to the herbicide Boxer 800 EC:

▪ sensitive weeds: *Stellaria media* (L.) Vill., *Lamium purpureum* L., *Chenopodium album* L., *Apera spica-venti*, *Veronica hederifolia*, *Veronica persica*, *Galium aparine* L.;
▪ moderately sensitive weeds: *Viola arvensis* Murr., *Papaver rhoeas* L., *Fallopia convolvulus* (L.) Á. Löve, *Matricaria chamomilla* L., *Poa annua L*.;
▪ resistant weeds: *Panicum crus galli* L.

### 4.3. Characteristics of Polymers

To neutralize the potential negative effects of the herbicide Boxer 800 EC on the biological properties of the soil, two pollutant-binding substances belonging to hydrogels were used:

▪ Sodium alginate (SA)—a solid substance with the molecular formula (NaC_6_H_7_O_6_) that occurs as a white powder with a pH of 5.5—8.0 and is produced by Agnex (Białystok, Poland). Its molecular weight ranges between 300 and 350 g mol^−1^. It is obtained from brown algae washed up on the Atlantic shore. It swells easily and binds water effectively. It has gelling properties at lower temperatures and reacts with calcium chloride to form a harder and stronger gel structure.
▪ Sodium polyacrylate (SP)—an organic chemical compound with the molecular formula (C3H_3_NaO_2_)_n_ that occurs as white granules characterized by a very high capacity to bind significant amounts of water. It stores water in the form of crystals and slowly releases it into the environment. It is produced by Biomus (Lublin, Poland) with the following characteristics: density: 700–800 kg m^−3^; water absorption capacity for distilled water: up to 20 g of water per 1 g of gel.

### 4.4. Characteristics of the Cultivated Plant

*Triticum aestivum* L. is one of the most important crops in the world and the main food source for about 35.0% of the global population [20]. It provides approximately 20.0% of the world’s calories and protein [22,86]. The adaptation of wheat to changing conditions can be achieved by modifying its phenotypic traits [87]. The morphological traits of *Triticum aestivum* L. vary depending on the species composition of wheat varieties, while phenotypic and morphological traits are important for varietal diversity [23,86].

In this trial, the cultivated crop was spring wheat (*Triticum aestivum* L.) of the variety “KWS Dorium C1”, produced by KWS SAAT SE & Co. KGaA (Einbeck, Germany). This variety has a medium ear-emergence date and is intended for late-autumn sowing or traditional spring sowing. It is characterized by a high yield potential, good resistance to fungal attack, and a favorable health profile. This variety shows very good resistance to mildew and brown rust. It is moderately resistant to yellow rust, brown leaf spot, leaf septoria, and ear fusarium.

### 4.5. Establishment of and Procedure for Conducting the Experiment

The vegetation experiment was established in May 2024 in a vegetation hall on the grounds of the University of Warmia and Mazury in Olsztyn (northeastern Poland). The pot experiment was conducted with four replicates per treatment across three series: I—soil with the addition of the herbicide Boxer 800 EC; II—soil with the addition of the herbicide and sodium alginate; III—soil with the addition of the herbicide and sodium polyacrylate. The herbicide Boxer 800 EC was applied to the soil once, in the form of an aqueous suspension, in the following amounts of active substance (mg kg^−1^ d.m. soil): 0.00 (control soil), 0.80 mg (recommended dose), 0.48 mg (dose 6-fold higher than the recommended dose), and 48.00 mg (dose 60-fold higher than the recommended dose). Literature data [17,20,88,89] indicate that the active substance prosulfocarb may pose a potential threat to organisms and may be toxic to humans and animals due to its properties, such as volatility in the atmosphere, mobility, and persistence in soil, water, and sediments. Against this background, the study not only examined the effects of the dose recommended by the manufacturer of the herbicide Boxer 800 EC on the biological properties of the soil, but also the effects of doses many times higher than the manufacturer’s recommendation. To neutralize the potential negative effects of the herbicide on soil microbiota and spring wheat, sodium alginate and sodium polyacrylate were additionally incorporated into the soil material in solid form in designated treatments at a rate of 6 g kg^−1^ d.m. soil. The hydrogel dose was determined based on literature data [82,83,84,85]. Adjuik et al. [90] report that hydrogel doses ranging from 2.0 to 8.0 g kg^−1^ soil were used in scientific studies. Albalasmeh et al. [91], Sepehri et al. [92] and Abdelghafar et al. [93] noted that plant productivity and improvements in the physical and chemical properties of soil increased with higher hydrogel content. To achieve optimal improvement in soil and plant quality, hydrogels were applied at a rate of 6 g per kg of dry soil. A total of 48 pots were used in the experiment. During the experiment, which was from May to July, the following atmospheric conditions prevailed: an average temperature of 16.23 °C, average humidity of 68.0%, and average solar radiation of 10.37 h per day.

The experiment was carried out as follows:

1. A quantity of 3.4 kg of soil, previously sieved through a mesh with a diameter of 5 mm, was weighed into plastic pots (capacity 3.5 dm^3^).
2. Fertilizer, the herbicide Boxer 800 EC, sodium alginate, and sodium polyacrylate were then applied to the soil in the appropriate amounts.
3. The soil was fertilized according to the nutrient requirements of *Triticum aestivum* L., in terms of pure components in mg kg^−1^ of soil dry matter: N—130 mg in the form of CO(NH_2_)_2_; P—60 mg in the form of KH_2_PO_4_, K—90 mg in the form of KH_2_PO_4_ + KCl; Mg—25 mg in the form of MgSO_4_ × 7H_2_O.
4. The contents of the pots were thoroughly mixed, and the soil was sown with spring wheat, with 20 grains per pot. The whole mixture was brought to a moisture content of 50% of the capillary water capacity using deionized water.
5. After the plants had germinated (7 days after sowing the spring wheat), 12 plants were left in each pot.
6. Throughout the experiment, soil moisture was replenished 3 times per day with deionized water.
7. Before harvesting the plants, the SPAD index (SPAD) was measured, while the length of the above-ground parts and ears of wheat was measured on the final day of the experiment (on the 50th day). After performing the plant biometric measurements, plant material was collected from each pot, and the fresh weight was determined.
8. The plant roots were extracted from the soil, the entire soil was thoroughly homogenized, and 700 g of fresh soil was taken from each combination for microbiological analysis, while 800 g was taken for physicochemical analysis.

### 4.6. Physicochemical Analyses of Soil

Standard methods were used to carry out the physicochemical analyses of the soil. Particle size distribution was determined using a Malvern Mastersizer 3000 laser diffraction analyzer (Malvern, Worcestershire, UK). Soil pH in 1 mol KCl dm^−3^ was measured using the potentiometric method. Hydrolytic acidity (HAC) and total exchangeable base cation (EBC) contents were determined by Kappen’s method, while organic carbon (C_org_) and total nitrogen (N_tot_) contents were measured using a Vario Max Cube CN analyzer (Elementar Analysen-Systeme GmbH, Langenselbold, Germany). The sorption capacity of the soil (CEC) and the degree of saturation of the soil with base cations (BS) were calculated based on the HAC and EBC values obtained. A detailed description of the analysis procedure can be found in Wyszkowska et al. [94] and Komorek et al. [95].

### 4.7. Determination of Microbial Numbers

The classical method, i.e., the serial dilution method, was used to determine the number of organotrophic bacteria (Org) and actinobacteria (Act). The determination procedure consisted of weighing 10 g of soil in 90 cm^3^ of sterile physiological saline (0.85% NaCl), shaking on a shaker (120 rpm min^−1^) for 30 min, and then performing a series of soil dilutions for each combination (10^–5^ and 10^–6^ dilutions). The soil suspension was added to Petri dishes in 4 replicates. A soil extract medium, as described by Bunt and Rovira, was used for the cultivation of organotrophic bacteria, while a medium containing nystatin and actidione, as described by Küster and Williams, was used for the cultivation of actinobacteria. The prepared microbiological material was incubated at 28 °C for 10 days. Colonies of organotrophic bacteria and actinobacteria were counted daily, and the number of microorganisms, expressed in colony-forming units (CFUs) per kilogram of soil dry matter, was calculated [96]. Based on the number of microorganisms, two indices were calculated: the colony development index (CD), according to the formula of Sarathchandra et al. [97], and the ecophysiological diversity index of microorganisms (EP), according to the formula of De Leij et al. [98]. The formulas for these indices are presented in the work of Lipińska et al. [99] and Baćmaga et al. [100].

### 4.8. Metagenomic Analysis of the Soil

The next-generation sequencing (NGS) method was used to identify bacteria. Bacterial genomic DNA was isolated from 1 g of soil (A&A Bio-Technology, Gdansk, Poland) using a modified MagnifiQ™ 1 Genomic DNA Instant Kit (A&A Bio-Technology). Mechanical lysis of the samples was performed in a FastPrep-24 device using zirconium beads. DNA purification was also carried out using the anti-inhibitor kit (A&A Bio-Technology). The concentration of genomic DNA was measured with a Qubit 4 fluorometer using a fluorimetric method. The presence of DNA in the samples was confirmed by real-time PCR performed in a CFX Connect thermal cycler (Biorad, Hercules, CA, USA) using SYBR Green dye as a fluorochrome. The universal primers 1055F (5′-ATGGCTGTCGTCAGCT-3′) and 1392R (5′-ACGGCGGTGTGTAC-3′) were used to amplify the 16S rRNA region [94]. The parameters of the isolated genomic DNA are listed in Table 9.

The metagenomic analysis of the bacteria was performed based on the V3–V4 hypervariable region of the 16S rRNA gene. Specific primer sequences (341F and 785R) were used to amplify the selected region and to construct the libraries. PCR was carried out using the Q5 Hot Start High-Fidelity 2× Master Mix (New England Biolabs, Heidelberg, Germany) according to the manufacturer’s protocol. Sequencing was performed with a MiSeq instrument in paired-end (PE) technology, 2 × 300 nt, using the Illumina v3 kit (Illumina, San Diego, CA, USA). The initial automated data analysis was conducted on the MiSeq sequencer using MiSeq Reporter (MSR) v2.6 software, which included automatic demultiplexing of the samples and the generation of fastq files containing the raw data. The obtained sequences were subjected to bioinformatic analysis using the QIIME 2 software package based on the Silva 138 reference sequence database, enabling classification of the reads at all taxonomic levels. Additionally, the DADA2 package (https://github.com/benjjneb/dada2, accessed on 12 November 2024) was employed, which facilitated the distinction of biologically derived sequences from those newly generated during sequencing and allowed for the isolation of unique biological sequences, known as ASV (amplicon sequence variant) sequences. The bacterial 16S rRNA sequences have been deposited in the NCBI GenBank database https://www.ncbi.nlm.nih.gov/nuccore/?term=PV529160: PV529624[accn] (accessed on 21 April 2025) under the accession numbers PV529160–PV529624.

### 4.9. Determination of Plant Growth and Development

On the 50th day of the experiment, when the spring wheat was at the 89 BBCH stage of development (Biologische Bundesantalt, Bundessortenamt und Chemische Industrie), the following parameters were determined: dry matter yield (g kg^−1^), length of the above-ground parts (cm), the spike length of the spring wheat (cm), and the SPAD index (SPAD). The SPAD index was measured using a chlorophyll meter from Spectrum Technologies, Inc. (KONICA MINOLTA, Inc., Chiyoda, Japan). To determine the dry matter yield of spring wheat, the aerial parts of the plants were cut and dried at 65 °C in a Binder D-78532 dryer (Binder GmbH, Tuttlingen, Germany).

### 4.10. Statistical Analysis of the Results

The results of the study were statistically analyzed using Statistica 13.3 software [101] at a significance level of *p* ≤ 0.05. The normality of the distribution of the results was verified using the Shapiro–Wilk test. Homogeneous groups were identified using Tukey’s test at *p* ≤ 0.05. The percentage of observed variability (ŋ^2^) in the studied factors was calculated for the microbiological and physicochemical properties of the soil and the growth and development of spring wheat. All data regarding the genetic diversity of bacteria were presented in graphical form for OTUs ≥ 1.0% of the total number of OTUs. The dominant bacterial classes in the soil were visualized using principal component analysis (PCA). Based on the total number of bacterial OTUs, the Shannon–Wiener index and the Simpson index were calculated [102]. The phylogenetic relationships of the nucleotide sequences of bacteria isolated from the soil were determined based on the 16S rRNA gene sequences using MEGA 7 with the neighbor-joining (NJ) method and a bootstrap value of 1000 [103]. The bacterial phylum and class, as well as the Shannon–Wiener index and the Simpson index, were visualized using TBtools-II software version 2.202 [104]. Data on the unique and common soil bacterial genera in the soil were presented as a Venn diagram created with InteractiVenn software (https://www.interactivenn.net, accessed on 15 March 2025) [105]. The predicted physicochemical properties of the proteins of the bacterial types were evaluated in silico using the ProtParam (https://web.expasy.org/protparam, accessed on 3 March 2025) tool available on the ExPASy portal [106]. The structural formula of prosulfocarb was generated using ISIS-Draw MDL version 2.3 [107].

## 5. Conclusions

The conducted study demonstrated that prosulfocarb exerts a significant impact on soil biological activity, leading to changes in the structure and diversity of soil microbial communities. In particular, high doses of the herbicide (48.0 mg kg⁻¹) markedly reduced the abundance and diversity of organotrophic bacteria and actinomycetes, which may threaten the stability of soil ecosystems. Our results showed that the presence of this herbicide significantly influenced the bacterial community structure. Application of this herbicide stimulated the proliferation of representatives of Proteobacteriota, Chloroflexi, Cyanobacteria, Firmicutes, and Verrucomicrobiota while reducing the relative abundance of Acidobacteriota, Actinobacteriota, Armatimonadota, Bacteroidota, Bdellovibrionota, Gemmatimonadota, Myxococcota, and Planctomycetota. These changes suggest that prosulfocarb may selectively promote certain bacterial groups, potentially disrupting the soil’s microbial balance. Consequently, these findings underscore the need to consider the impact of agrochemicals on the soil microbiome when designing sustainable agroecosystem management strategies.

Prosulfocarb application adversely affected the growth and development of spring wheat, causing the inhibition of biomass accumulation and reductions in plant morphological parameters. These results indicate potential risks associated with the use of this herbicide in terms of plant health and agroecosystem productivity.

Furthermore, the addition of sodium alginate was shown to mitigate the negative effects of the herbicide, both through a direct influence on microbiological bioactivity and indirectly by modulating the bacterial community structure. Sodium alginate demonstrates potential as a carrier that supports soil microorganisms under chemical stress conditions, making it a promising component in bioremediation technologies.

From a practical perspective, these findings suggest that considering soil biological properties and applying biodegradable polymers may serve as valuable tools in agrochemical management. Developing integrated strategies that combine crop protection with the preservation of soil microbiomes can support sustainable agriculture and reduce the long-term environmental consequences of contamination.

## Figures and Tables

**Table 1:** Percentage of observed variability (ŋ^2^) of the investigated factors (%) on the microbiological properties of soil.

Variables	N_Org	N_Act	CD_Org	CD_Act	EP_Org	EP_Act
HD	17.014	1.653	41.347	10.436	10.592	25.558
TP	72.701	96.742	46.092	64.233	11.990	18.913
HD×TP	10.017	0.143	9.723	24.940	74.891	51.418
error	0.269	1.462	2.837	0.391	2.526	4.111

HD—herbicide dose; TP—type of polymer; N_Org—number of organotrophic bacteria; N_Act—number of actinobacteria; CD_Org—organotrophic bacteria colony development index; CD_Act—actinobacteria colony development index; EP_Org—ecophysiological diversity index of organotrophic bacteria; EP_Act—ecophysiological diversity index of actinobacteria.

**Table 2:** The number of organotrophic bacteria and actinobacteria in soil with herbicides and polymers (in cfu 10^9^ kg^−1^ d.m. of soil).

HD	Org			Act		
	C	SA	SP	C	SA	SP
0.00	3.536 ± 0.266 ^h^	18.006 ± 0.446 ^a^	4.232 ± 0.342 ^f^	2.536 ± 0.172 ^f^	9.866 ± 0.844 ^c^	2.411 ± 0.278 ^g^
0.80	5.480 ± 0.971 ^e^	16.383 ± 0.402 ^b^	3.849 ± 0.427 ^g^	3.731 ± 0.541 ^e^	11.431 ± 0.931 ^b^	2.255 ± 0.024 ^h^
4.80	3.054 ± 0.653 ^i^	11.785 ± 0.981 ^c^	2.949 ± 0.027 ^j^	2.467 ± 0.665 ^g^	12.537 ± 0.657 ^a^	1.934 ± 0.196 ^i^
48.0	1.417 ± 0.244 ^l^	8.200 ± 0.166 ^d^	2.049 ± 0.475 ^k^	1.700 ± 0.294 ^j^	9.205 ± 0.779 ^d^	1.097 ± 0.206 ^k^

HD—herbicide dose (mg kg^−1^ d.m. soil); Org—organotrophic bacteria; Act—actinobacteria; C—control soil; SA—soil with sodium alginate; SP—soil with sodium polyacrylate. Homogeneous groups (^a–l^) were determined separately for organotrophic bacteria and actinobacteria depending on the herbicide dose and polymer type. Different letters indicate statistically significant differences between groups at the *p* ≤ 0.05 level.

**Table 3:** The predicted physicochemical properties of the bacterial protein based on its nucleotide sequence were calculated using the ProtParam database on the ExPasy portal [31,32].

Genus	Aa	MW	pI	ExC	I_ix_	A_ix_	GRAVY
*Bryobacter*	427	33.639	5.07	6250	40.01	23.19	0.745
*Nocardioides*	407	31.996	5.08	5250	42.23	23.83	0.672
*Streptomyces*	402	32.133	5.08	5500	39.98	23.23	0.678
*Gaiella*	426	33.425	5.07	6125	44.43	24.41	0.754
*Conexibacter*	427	33.639	5.07	6250	40.01	23.19	0.745
*Flavisolibacter*	422	33.968	5.09	5250	41.14	27.73	0.717
*Terrimonas*	422	33.881	5.08	4875	42.07	28.67	0.704
*Nubsella*	422	33.731	5.09	5125	39.66	27.49	0.708
*Pedobacter*	422	33.669	5.08	5500	42.99	27.01	0.736
*Leptolyngbya*	405	31.798	5.09	4750	41.46	27.41	0.694
*Tumebacillus*	428	33.778	5.07	6125	44.94	23.83	0.742
*Bacillus*	428	33.753	5.08	5625	38.81	25.93	0.723
*Paenibacillus*	426	33.603	5.08	5375	33.55	26.29	0.712
*Gemmatimonas*	419	32.918	5.07	6125	46.61	21.28	0.699
*Reyranella*	402	31.625	5.09	5000	41.42	25.87	0.695
*Devosia*	402	31.773	5.09	5125	40.48	26.12	0.706
*Mesorhizobium*	402	31.827	5.09	5000	39.38	26.37	0.695
*Bradyrhizobium*	402	31.779	5.09	5250	40.90	25.37	0.704
*Pseudolabrys*	402	31.703	5.08	5500	40.83	24.63	0.729
*Sphingomonas*	428	33.866	5.08	5875	42.43	25.47	0.740
*Dyella*	427	33.646	5.08	5375	40.69	26.00	0.706
*Rhodanobacter*	427	33.698	5.09	5250	36.61	26.23	0.686
*Luteimonas*	427	33.634	5.08	5500	37.38	25.76	0.706
*Nitrspira*	427	33.742	5.08	5750	45.37	26.70	0.756
*Lacunisphaera*	426	33.858	5.08	5625	46.33	27.46	0.765

Aa—number of amino acids; MW—molecular weight (kDa); pI—isoelectric point; Początek formularza ExC—extinction coefficient (M^−1^ cm^−1^); I_ix_—instability index (I_ix_ > 40—protein is unstable; I_ix_ < 40—protein is stable); A_ix_—aliphatic index; GRAVY—grand average of hydropathicity.

**Table 4:** Percentage of observed variability (ŋ^2^) in the investigated factors (%) on the growth and development of spring wheat.

Variables	PDM	LAPP	PEL	SPAD
HD	82.047	98.919	99.349	98.211
TP	7.495	0.396	0.118	0.009
HD × TP	7.495	0.396	0.118	0.009
error	2.964	0.289	0.414	1.770

HD—herbicide dose; TP—type of polymer; PDM—plant dry matter; LAPP—length of above-ground parts of plants; PEL—plant ear length; SPAD—SPAD index.

**Table 5:** Growth and development of *Triticum aestivum* L.

HD	PDM	LAPP	PEL	SPAD
Soil without the addition of polymers (SWAP)				
0.00	25.750 ^a^	66.000 ^a^	8.000 ^b^	45.600 ^b^
0.80	25.970 ^a^	65.750 ^a^	8.125 ^a^	47.525 ^a^
4.80	21.633 ^b^	62.250 ^b^	8.250 ^a^	39.617 ^e^
48.0	0.000 ^g^	0.000 ^g^	0.000 ^f^	0.000 ^f^
Soil with the addition of sodium alginate (SA)				
0.00	18.165 ^c^	61.750 ^b^	7.750 ^c^	45.783 ^b^
0.80	16.815 ^d^	55.500 ^d^	8.000 ^b^	44.767 ^c^
4.80	6.508 ^f^	48.500 ^e^	7.750 ^c^	44.050 ^c^
48.0	0.000 ^g^	0.000 ^g^	0.000 ^f^	0.000 ^f^
Soil with the addition of sodium polyacrylate (SP)				
0.00	11.980 ^e^	59.250 ^c^	7.500 ^d^	44.758 ^c^
0.80	16.205 ^d^	54.000 ^d^	7.125 ^e^	45.433 ^b^
4.80	6.370 ^f^	40.500 ^f^	7.125 ^e^	40.475 ^d^
48.0	0.000 ^g^	0.000 ^g^	0.000 ^f^	0.000 ^f^

HD—herbicide dose (mg kg^−1^ d.m. soil); PDM—plant dry matter (g kg^−1^); LAPP—length of above-ground parts of plants (cm); PEL—plant ear length (cm); SPAD—SPAD index. Homogeneous groups (^a–g^) were determined separately for each plant parameter depending on the herbicide dose and polymer type. Different letters indicate statistically significant differences between groups at the *p* ≤ 0.05 level.

**Table 6:** Percentage of observed variations (ŋ^2^) in the studied factors (%) of the physicochemical properties of the soil.

Variables	C_org_	N_tot_	C/N	pH	HAC	EBC	CEC	BS
HD	23.389	48.895	17.377	31.481	22.325	15.004	15.917	11.838
TP	13.465	16.642	31.543	43.519	46.225	73.354	63.586	73.746
HD × TP	62.324	32.173	49.130	23.222	29.244	7.932	17.572	11.275
error	0.822	2.289	1.951	1.778	2.206	3.710	2.924	3.140

HD—herbicide dose (mg kg^−1^ d.m. soil); TP—type of polymer; C_org_—organic carbon content (g kg^−1^ d.m. soil); N_tot_—nitrogen carbon content (g kg^−1^ d.m. soil); C/N—ratio of organic carbon content to total nitrogen content; pH—soil pH in 1 M KCl dm^−3^; HAC—hydrolytic acidity (mmol^+^ kg^−1^ d.m. soil); EBC—sum of exchangeable bases (mmol^+^ kg^−1^ d.m. soil); CEC—sorption capacity (mmol^+^ kg^−1^ d.m. soil); BS—base saturation (%).

**Table 7:** Basic physicochemical properties of the soil.

HD	C_org_	N_tot_	C/N	pH	HAC	EBC	CEC	BS
Soil without the addition of polymers (SWAP)								
0.00	7.575 ^d^	1.285 ^a^	5.896 ^h^	4.725 ^e^	15.375 ^d^	29.000 ^h^	44.375 ^h^	65.220 ^g^
0.80	7.210 ^f^	1.120 ^e^	6.437 ^f^	4.775 ^d^	15.750 ^c^	29.500 ^g^	45.250 ^g^	65.206 ^g^
4.80	7.110 ^g^	1.060 ^f^	6.708 ^d^	4.725 ^e^	16.125 ^b^	30.000 ^f^	46.125 ^f^	65.044 ^g^
48.0	6.715 ^h^	1.050 ^f^	6.398 ^g^	4.700 ^f^	16.500 ^a^	29.500 ^g^	46.000 ^f^	64.136 ^h^
Soil with the addition of sodium alginate (SA)								
0.00	7.130 ^g^	1.095 ^f^	6.517 ^e^	4.825 ^b^	15.375 ^d^	32.500 ^e^	47.875 ^d^	67.888 ^f^
0.80	7.310 ^e^	1.130 ^d^	6.467 ^f^	4.825 ^b^	15.750 ^c^	33.000 ^d^	48.750 ^d^	67.703 ^f^
4.80	7.515 ^d^	1.130 ^d^	6.668 ^d^	4.825 ^b^	15.375 ^d^	34.500 ^c^	49.875 ^d^	69.172 ^e^
48.0	8.045 ^c^	1.120 ^e^	7.222 ^c^	4.775 ^d^	14.625 ^f^	35.500 ^b^	50.125 ^b^	70.822 ^c^
Soil with the addition of sodium polyacrylate (SP)								
0.00	9.545 ^a^	1.225 ^b^	7.797 ^a^	4.875 ^a^	15.000 ^e^	34.500 ^d^	49.500 ^e^	69.710 ^d^
0.80	8.825 ^b^	1.210 ^c^	7.318 ^b^	4.875 ^a^	14.663 ^f^	35.000 ^c^	49.663 ^e^	70.478 ^c^
4.80	7.375 ^e^	1.115 ^e^	6.619 ^e^	4.825 ^b^	14.288 ^g^	35.500 ^b^	49.788 ^c^	71.301 ^b^
48.0	6.560 ^i^	1.040 ^f^	6.324 ^g^	4.800 ^c^	13.913 ^h^	36.500 ^a^	50.413 ^a^	72.405 ^a^

HD—herbicide dose (mg kg^−1^ d.m. soil); C_org_—organic carbon content (g kg^−1^ d.m. soil); N_tot_—nitrogen carbon content (g kg^−1^ d.m. soil); C/N—ratio of organic carbon content to total nitrogen content; pH—soil pH in 1 M KCl dm^−3^; HAC—hydrolytic acidity (mmol^+^ kg^−1^ d.m. soil); EBC—sum of exchangeable bases (mmol^+^ kg^−1^ d.m. soil); CEC—sorption capacity (mmol^+^ kg^−1^ d.m. soil); BS—base saturation (%). Homogeneous groups (^a–i^) were calculated separately for each soil physicochemical parameter depending on the herbicide dose and polymer type. Different letters indicate statistically significant differences between groups at the *p* ≤ 0.05 level.

**Table 8:** Characterization of the active substance prosulfocarb [85].

Prosulfocarb	Parameter		Formula/Value
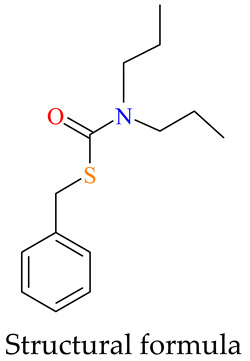	Chemical formula		C_14_H_21_NOS
	Substance groups		thiocarbamate
	Solubility in water at 20 °C (mg dm⁻^3^)		13.2
	Solubility in organic solvents at 20 °C (mg dm⁻^3^)		250,000 (acetone)
	Vapor pressure at 20 °C (mPa)		0.79
	Octanol–water partition coefficient at pH 7, 20 °C	P	3.02 × 10^4^
		Log P	4.48
	Soil degradation aerobic (days)	DT_50_ (typical)	11.9
		DT_50_ (lab at 20 °C)	11.9
		DT_50_ (field)	9.8
	K_f_ (cm^3^ g^−1^)		23.1
	K_foc_ (cm^3^ g^−1^)		1693

**Table 9:** The concentration of isolated genomic DNA.

Parameter	C	B	SA	B + SA	SP	B + SP
DNA concentration (µg cm^−3^)	15.1	8.96	11.10	6.74	11.9	14.6

C—control soil; B—soil contaminated with the Boxer 800 EC herbicide; SA—soil supplemented with sodium alginate; B + SA—soil contaminated with herbicide and supplemented with sodium alginate; SP—soil supplemented with sodium polyacrylate; B + SP—soil contaminated with herbicide and supplemented with sodium polyacrylate.

## Data Availability

The data presented in this study are available on request from the corresponding author.

## Supplementary Materials

The following supporting information can be downloaded at: https://www.mdpi.com/article/10.3390/ijms26125452/s1.
