# Supplementary material for: Influence of Prosulfocarb and Polymer Supplementation on Soil Bacterial Diversity in Triticum aestivum L. Cultivation
Source: Int J Mol Sci. 2025 Jun 6;26(12):5452. doi: 10.3390/ijms26125452 (PMC12193064; doi:10.3390/ijms26125452)
Supplement: Supplementary file 1 [file ijms-26-05452-s001.zip › ijms-3642084-supplementary.pdf]

International Journal of
Molecular Sciences

MDPI

Supplementary Materials

Influence of Prosulfocarb and Polymer Supplementation on Soil Bacterial Diversity in *Triticum aestivum* L. Cultivation

Małgorzata Baćmaga, Jadwiga Wyszkowska * and Jan Kucharski

Department of Soil Science and Microbiology, Faculty of Agriculture and Forestry, University of Warmia and Mazury in Olsztyn, Łódzki 3 Sq., 10-719 Olsztyn, Poland
* Correspondence: jadwiga.wyszkowska@uwm.edu.pl

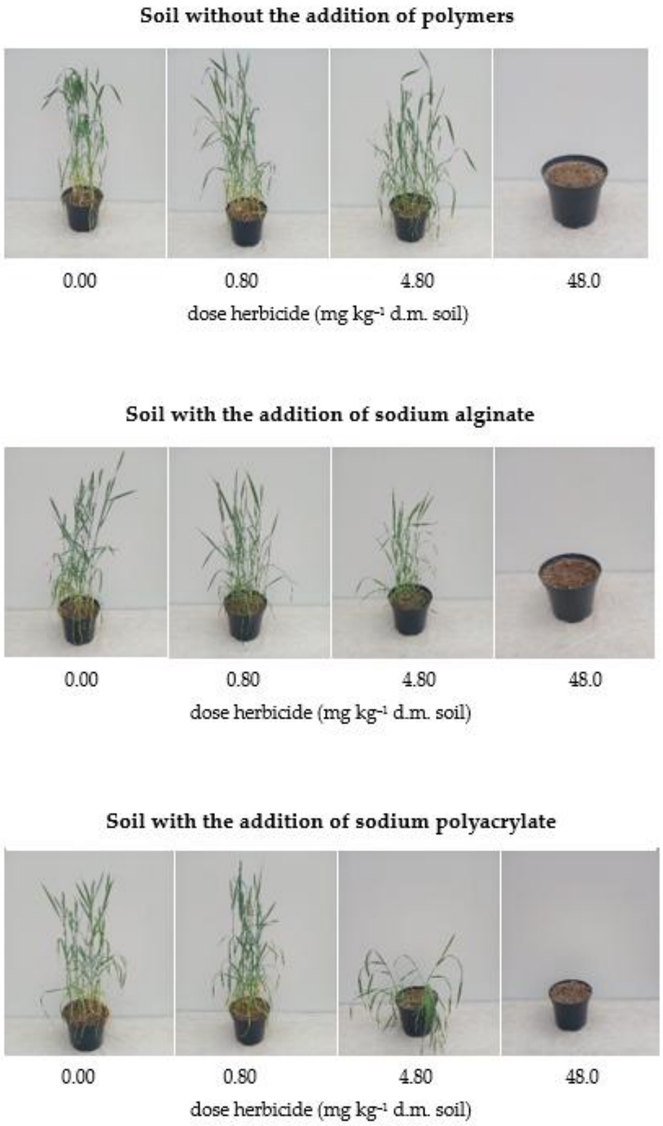

Figure S1. Growth and development of *Triticum aestivum* L. (89 BBCH) in soil treated with Boxer 800 EC herbicide and supplemented with sodium alginate and sodium polyacrylate.
